# Case Report: Family Curse: An *SCN5A* Mutation, c.611C>A, p.A204E Associated With a Family History of Dilated Cardiomyopathy and Arrhythmia

**DOI:** 10.3389/fcvm.2022.822150

**Published:** 2022-05-06

**Authors:** Wen Huang, Rui Xu, Ning Gao, Xia Wu, Cong Wen

**Affiliations:** ^1^Department of Medical Ultrasound, Shandong Provincial Qianfoshan Hospital, Shandong University, Jinan, China; ^2^Department of Cardiology, The First Affiliated Hospital of Shandong First Medical University & Shandong Provincial Qianfoshan Hospital, Shandong Medicine and Health Key Laboratory of Cardiac Electrophysiology and Arrhythmia, Shandong University, Jinan, China; ^3^Department of Cardiology, Shandong Provincial Qianfoshan Hospital, Weifang Medical University, Jinan, China

**Keywords:** *SCN5A*, *PRKAG2*, dilated cardiomyopathy, sudden cardiac death, familial case report

## Abstract

**Objective:**

We report a 3-generation family with *SCN5A* c.611 C>A rare variant, whose clinical characteristics are dilated cardiomyopathy (DCM) combined with multifocal ectopic Purkinje-related premature contractions (MEPPC). We tried to explain why the same *SCN5A* variant carriers had different phenotypes.

**Methods:**

We collected the clinical data from the family, and followed up this family members. Genetic testing was done for whom DNA samples could be collected.

**Results:**

Information was collected from 15 people in this family, 8 of whom had genetic testing. The *SCN5A* variant was present in all patients of this family, whose clinical features showed DCM combined with MEPPC. The proband's children developed DCM and MEPPC in their childhood. They both carried a *SCN5A* p.A204E mutation from their mother and a mutation *PRKAG2* p.D372N from their father. The son did heart transplant and his heart was both dilated and thickened. The pathology confirmed the presence of glycogen accumulation in the myocardium, which were consistent with the diagnosis of *PAKAG2* syndrome.

**Conclusion:**

SCN5A c.611 C>A variant was related to DCM combined with MEPPC. This case report is the first to demonstrate that a combination of *SCN5A* and *PRKAG2* mutations can cause DCM plus MEPPC and *PRKAG2* Syndrome.

## Introduction

Dilated cardiomyopathy (DCM) is the most common non-ischemic cardiomyopathy, with an incidence of about 1/2500, which can lead to severe heart failure. It is associated with arrhythmia and sudden death, and the incidence of sudden death within 5 years is 10–15% (1). The main pathogenic genes are related to cardiac structural proteins, sarcomereproteins, and nuclear membrane proteins. About 2% of patients with heart dilatation are caused by *SCN5A* gene mutation.

**Figure 1:**
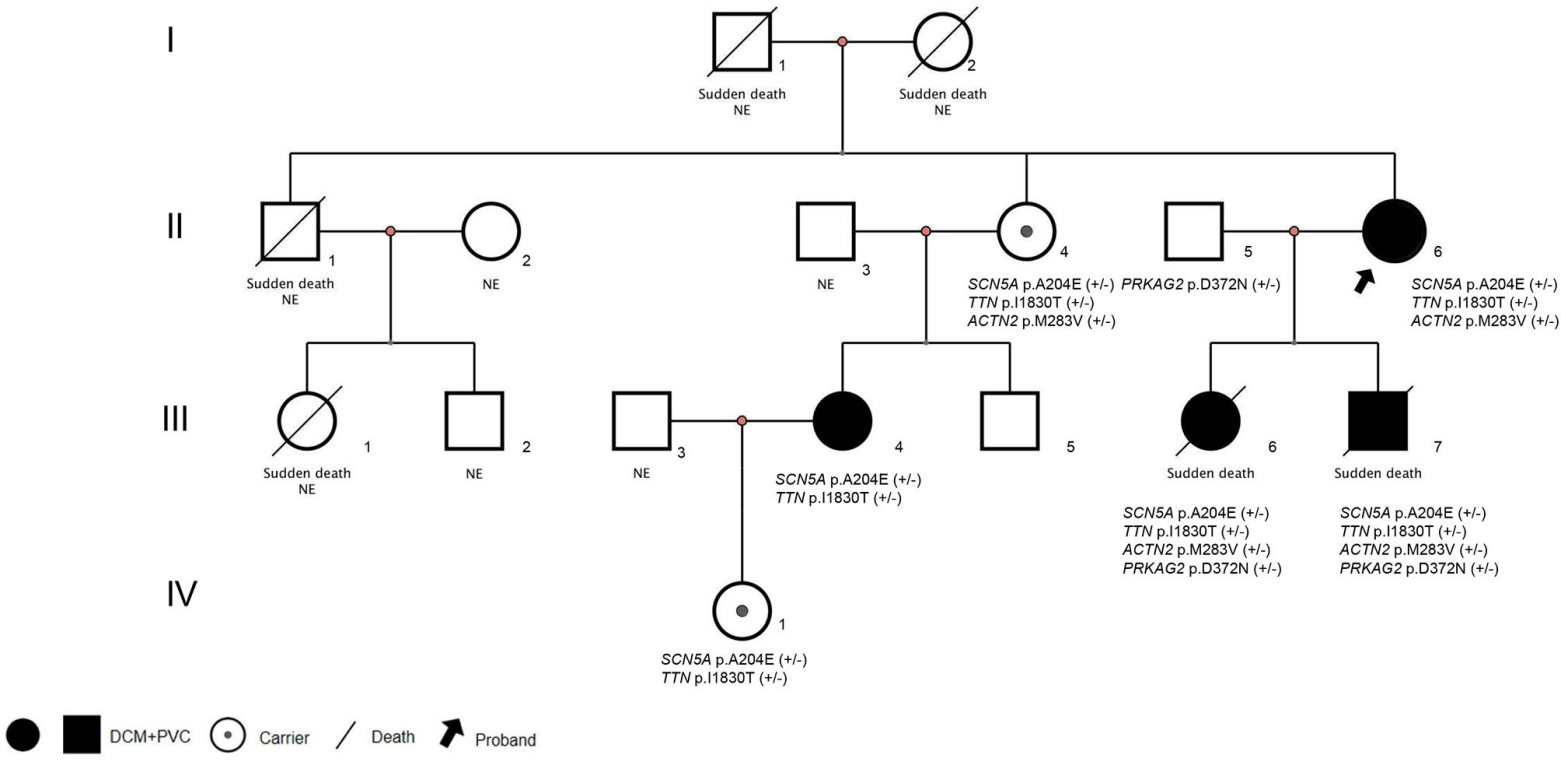
Pedigrees and diagnosis of the families assessed. Squares indicate male family members, circles indicate female family members. NE:Genomic DNA was not examined. (+/-): Haplotype. DCM, Dilated cardiomyopathy; PVC, Premature ventricular contractions.

A family case of DCM with an *SCN5A* mutation, c.611C>A, p.A204E is reported as follows. The same mutation has been reported in one case in the literature, and no family report has been found (2). The variant is unique from, so far, reported typical *SCN5A* variants because of its characteristic phenotype expression: Purkinje-related premature contractions. We report a 3-generation family with this rare variant (Figure 1), where patients presented dilated cardiomyopathy (DCM) combined with premature ventricular contractions (PVC), and carriers manifested a variety of premature contractions.

## Case Presentations

### Case 1

**Figure 2:**
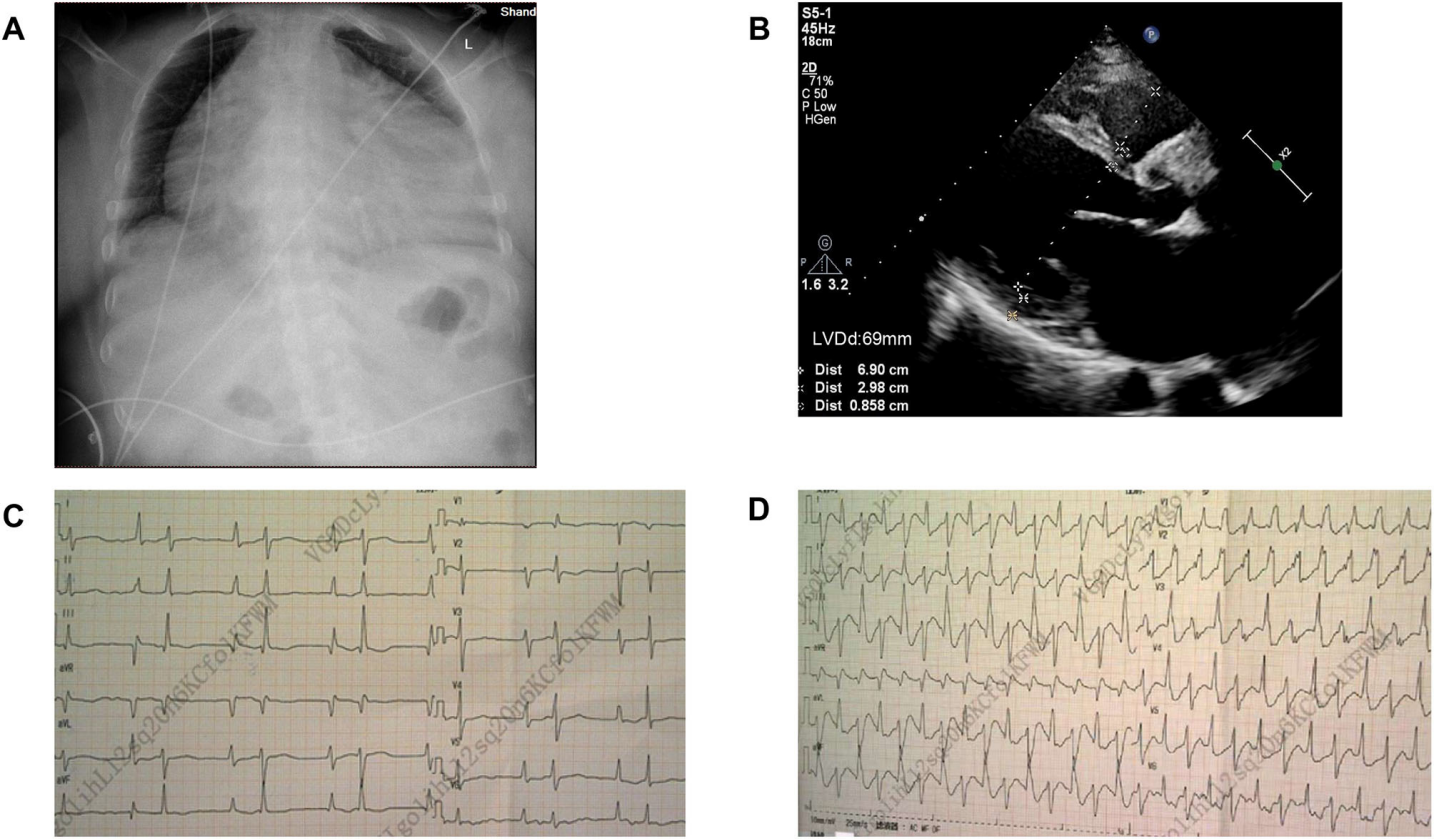
Image of case 1 (Figure 1:II-6). **(A)** Chest X-ray showed congestion with a cardiothoracic ratio of 80%. **(B)** Echocardiogram revealed an enlarged left ventricle. LVDd, Left ventricular end-diastolic diameter. **(C,D)**: Electrocardiogram (ECG) comparison between the normal state and frequent premature ventricular contractions.

**Figure 3:**
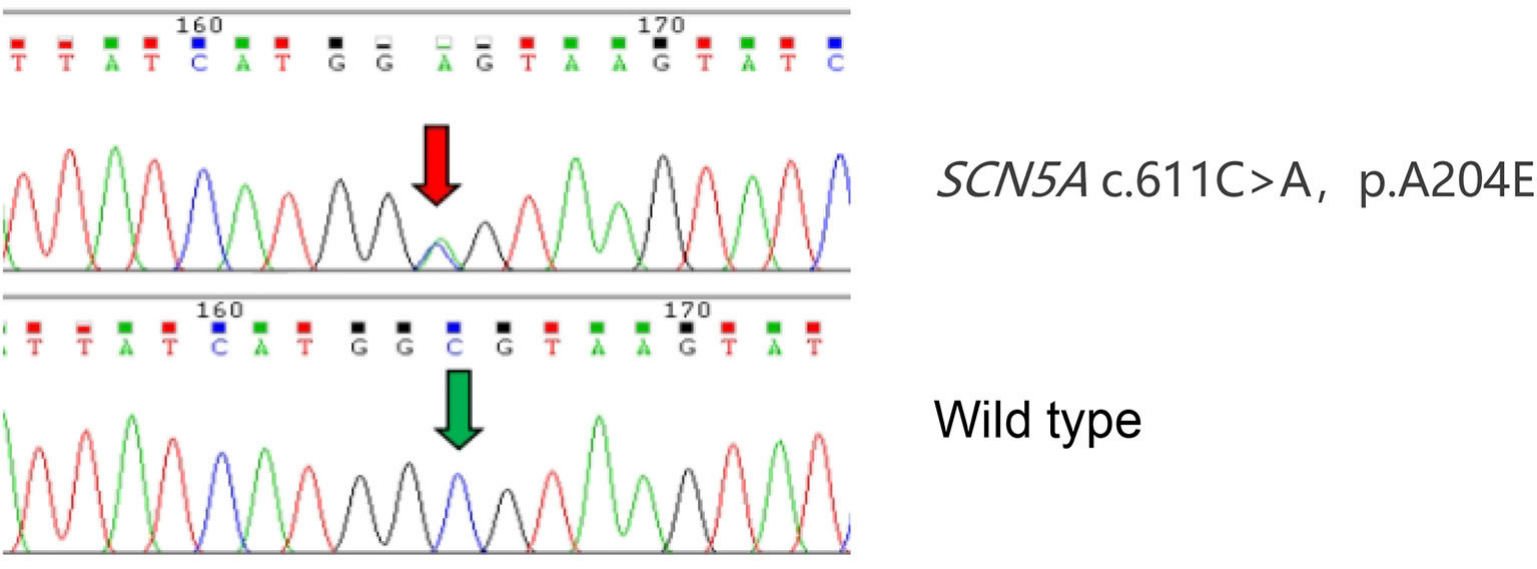
Identification of the SCN5A variant. Sanger sequence SCN5A in a wild-type (WT) subject and proband confirms the presence of the A204E variation. The variant nucleotide residue is indicated by a red arrow. The same nucleotide residue of WT is indicated by a green arrow.

The proband (Figure 1: II-6), a 49-year-old woman, was admitted to a hospital with a 14-year history of recurrent chest distress, aggravated by palpitations for 4 days. She had a family history of sudden death: her father (I-1) died of cerebrovascular disease at 50 years, her mother (I-2) died suddenly at 40 years, a brother (II-1) died suddenly at 33 years old, and his daughter (III-1) died suddenly at 19 years. All of the sudden deaths happened during sleep. The patient had a daughter (III-6) and a son (III-7), who were diagnosed with DCM at the age of 16 and 9 years, respectively. The patient developed chest tightness and dyspnea, accompanied by nausea and vomiting after catching a cold 14 years ago. At that time, she underwent cardiac color ultrasound and was diagnosed with DCM. Hospitalization, ventricular tachycardia, and ventricular fibrillation occurred several times. Twelve years ago, 1 month after having her second child, the proband was readmitted for chest tightness for about 3 weeks, aggravated by palpitations for 1 week. During her hospital stay, more than 50 episodes of ventricular tachycardia or ventricular fibrillation occurred; an implantable cardiac defibrillator was recommended, but the patient refused. After discharge, the patient took metoprolol and was followed regularly. A conventional echocardiogram showed a large left ventricular end-diastolic diameter (LVEDD) of 69 mm and an LV ejection fraction (LVEF) of 40% (Figure 2). Because of her family history, genomic DNA was extracted from the peripheral blood of eight family members [the proband and her husband (II-5), two children (III-6) (III-7), sister (II-4), the sister's two children (III-4) (III-5), and granddaughter (IV-1)], and a genomic library was constructed. The enriched target gene fragments were sequenced by Illumina sequencing (3). The proband carried 3 possible pathogenic mutation associated with DCM and PVC, including *SCN5A* p.A204E, *TTN* p.I1830T, and *ACTN2* p.M283V. The pathogenicity of the mutation was analyzed concerning the American College of Medical Genetics and Genomics(ACMG)guidelines (4). *SCN5A* p.A204E was considered “likely pathogenic.” *TTN* p.I1830T and *ACTN2* p.M283V were considered “uncertain significance.” Figure 3 shows the *SCN5A* variant of the DNA analyses. The proband's parents, brother, and niece were not tested because they had all died before this time point. Her brother's ex-wife (II-2) and son (III-2) were not tested because the ex-wife took her son when she remarried and lost contact.

### Case 2

At 16 years, the proband's daughter (III-6) was admitted to a hospital for “chest tightness and shortness of breath for 20 days.” Twenty days after catching a cold, she developed fatigue, chest congestion, shortness of breath, and gradually decreased endurance. She became short of breath after walking for 5–6 min and could not lie supine at night. Twelve days later, an outpatient electrocardiogram (ECG) showed frequent atrial premature beats accompanied by differential transmission and interface escape. The echocardiogram showed a large LV, and the LVEF was 24%. She had previously been healthy and had a normal ECG when she was 13. She died suddenly at the age of 19 years from “shock,” following her brother's car accident. In addition to carrying the same three mutations as her mother, Case 2 carried the *PRKAG2* p.D372N variant from her father (II-5).

### Case 3

**Figure 4:**
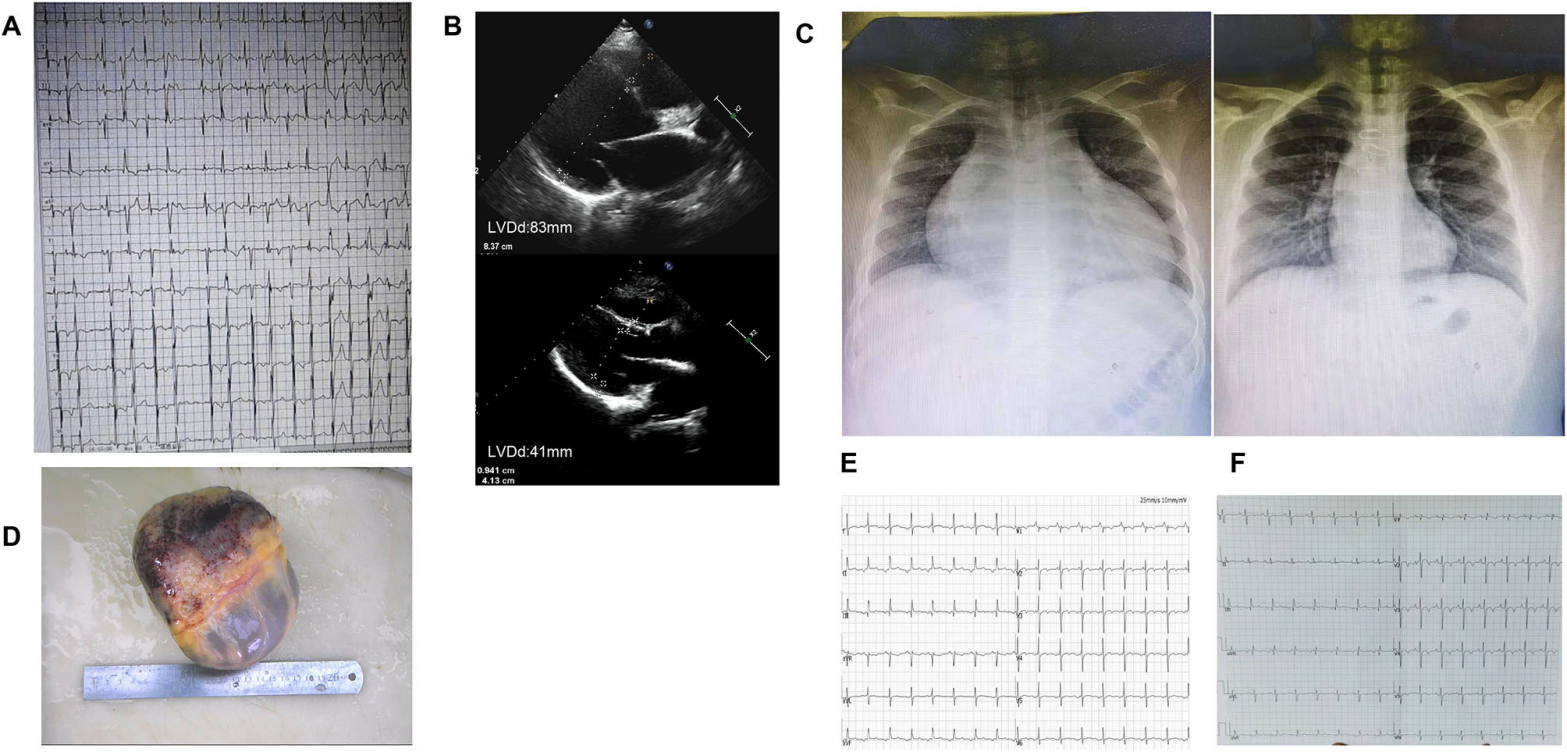
Image of case 3 (III-7). **(A)** ECG before heart transplantation showed frequent ventricular premature contractions with narrow QRS. **(B)** Echocardiogram findings of changes in heart size before and after heart transplantation. **(C)** X-ray findings of changes in heart size before and after heart transplantation. **(D)** The enlarged specimen of the heart. **(E,F)** ECG in the first and second years after heart transplantation,T wave was inverted.

**Figure 5:**
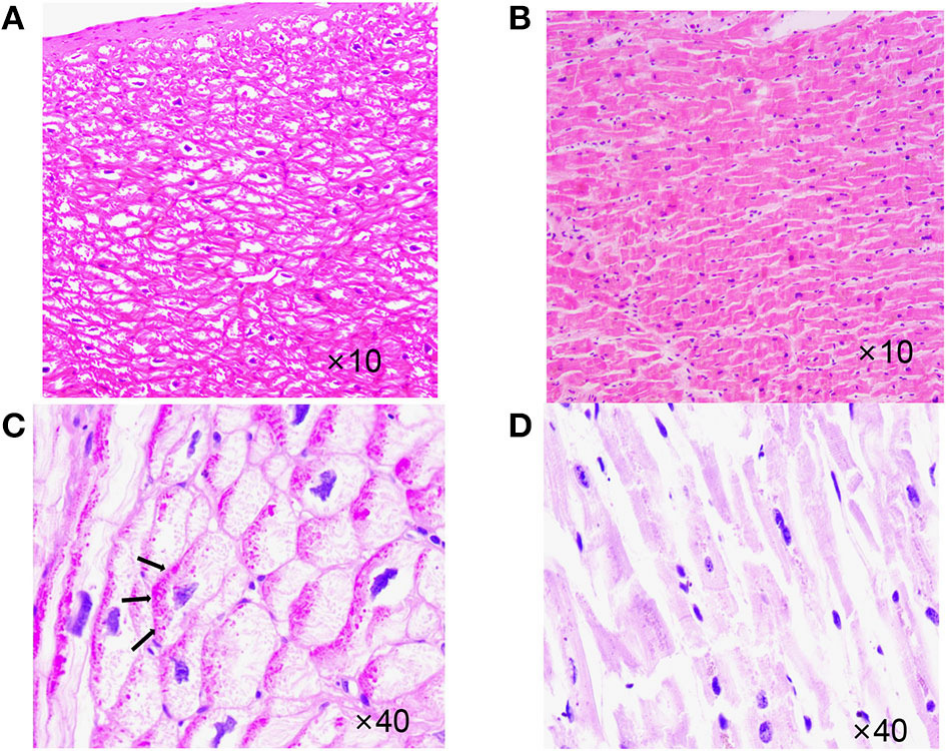
Case 3 (III-7) vs. normal myocardial tissue. **(A)** Hematoxylin-eosin staining (HE staining),cardiomyocyte hypertrophy with large, malformed nuclei,some myocardial cells had vacuolated cytoplasm. **(B)** HE staining,Normal myocardial tissue. **(C)** Periodate-Scherff staining (PAS staining), red PAS staining-positive material in the cytoplasm of cardiomyocytes. Arrows indicate intracellular glycogen accumulation. **(D)** PAS staining, Normal myocardial tissue, red PAS staining-negative.

The proband's son (III-7), her second child, was hospitalized with a fractured femur from a car accident at 6 years. At that time, echocardiography showed no abnormality. Three years later, he was hospitalized for nausea and vomiting for 4 days and chest distress for 2 days after catching a cold. An ECG showed multi-source ventricular-accelerated spontaneous rhythm, nodal beats, nodal tachycardia, atrial chaotic tachycardia, ventricular concurrent rhythm, a visible ventricular fusion wave, and ST-T changes. Echocardiography showed an enlarged heart, and the LVEF was 27%. The diagnosis was DCM, arrhythmic cardiomyopathy, and heart failure. A pediatrician diagnosed the boy with end-stage heart failure, and heart transplantation was performed in our hospital when he was 10. His heart was significantly expanded before surgery, and a chest X-ray revealed slight congestion, with a cardiothoracic ratio of 80%. The LVEDD was 83 mm, and the LVEF was 24% (Figure 4). His heart was found to have increased marked dilatation of four chambers and thickened ventricular walls, interventricular septum thickness (IVST) 16 mm and posterior wall thickness (PWT) 20 mm. The changes we can see under the microscope are hypertrophy of cardiomyocytes with large and malformed nuclei. Some myocardial cells had vacuolated cytoplasm. The periodate-Scherff staining (PAS staining) found red PAS staining-positive material in the cytoplasm of cardiomyocytes (Figure 5).

Re-examination within 2 years after surgery showed a normal heart size; the LVEDD was 41 mm, and the LVEF was 74% (Table 1). Two and a half years after heart transplantation, he died suddenly at school, although nothing abnormal was found.

**Table 1:** The change of echocardiogram index and BNP of case 3 (III-7).

**AGE(year)**	**LVDd(mm)**	**IVST(mm)**	**PWT(mm)**	**LVEF(%)**	**BNP (pg/ml)**
6	36	6	5	60	
9	54	7.5	6	27	9,930
9.5	83	7	6	24	1,380
10	84	7	6	23	2,040
**Heart transplantation**					
10	44	9	8	60	123
11	41	9	8	64	12.2
11.5	39	9	8.5	77	6.8

*LVDd, Left ventricular end-diastolic diameter; IVST, Interventricular septum thickness; PWT, Posterior wall thickness; LVEF, Left ventricular ejection fraction; BNP, Brain natriuretic peptide*.

The boy did the gene test before the heart transplantation. He carried multiple mutations, including *SCN5A* p.A204E, *RMND1* frame-shift mutation, *MLYCD* p.R412C, *POMT1* p.Y282C, *ACTN2* p.M283V, *TTN* p.I18305T, and *PRKAG2* p.D372N. Except for *SCN5A* p.A204E, the ACMG guidelines considered its pathogenicity to be “likely pathogenicity,” and other variants were considered to be of “uncertain significance.”

### Case 4

**Figure 6:**
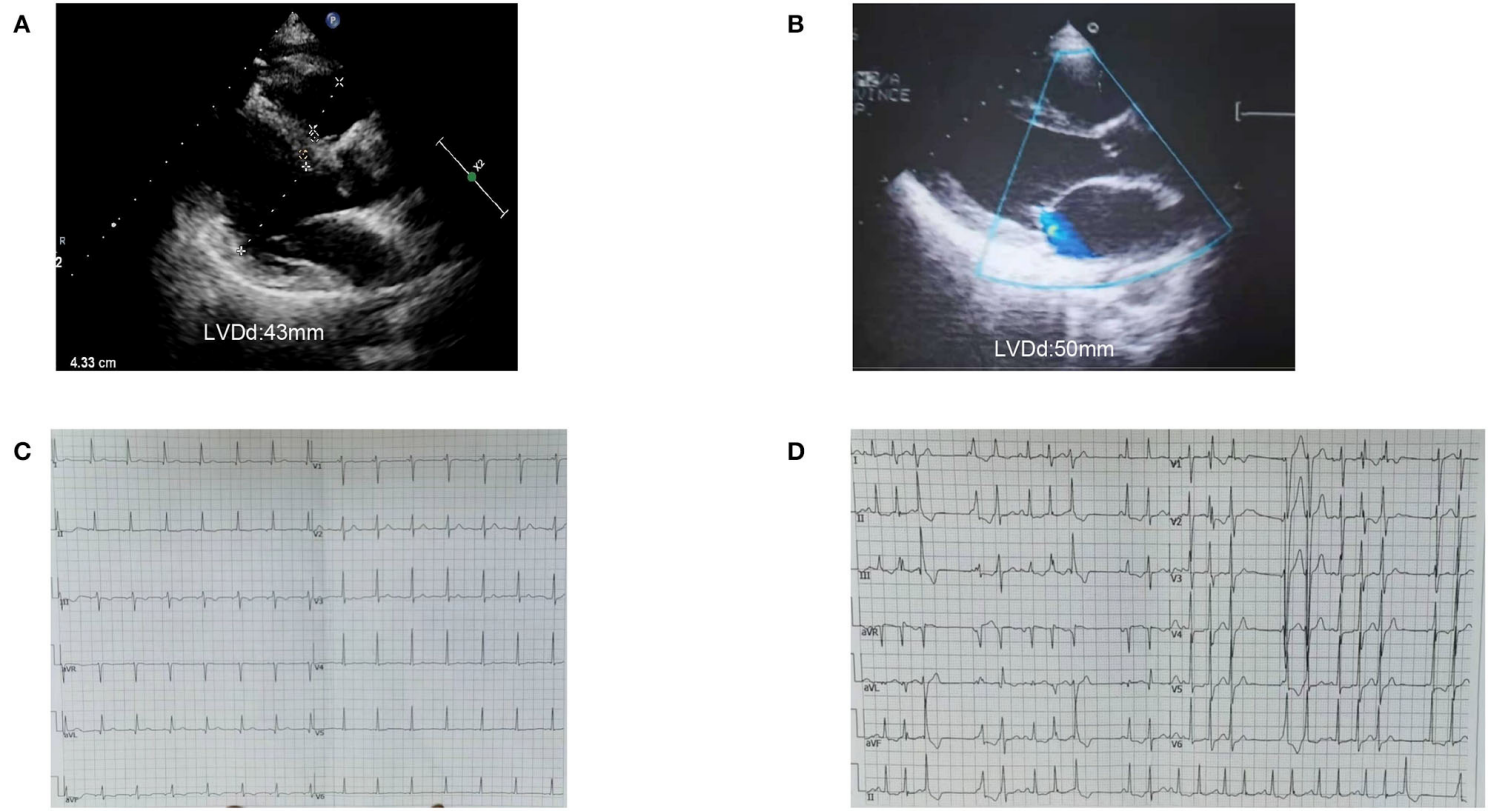
Image of case 4 (II-4) and 5 (III-4). **(A,B)** Case 4 and Case 5 presented a normal left ventricle on echocardiogram. **(C)** ECG of case 4 (II-4) showed atrial rhythm, PR was shorter than 0.12s. **(D)** ECG of case 5 (III-4) showed frequent ventricular premature contractions with narrow QRS.

The proband's sister (II-4) is an asymptomatic carrier of the *SCN5A* mutation, c.611C>A, p.A204E and is in good health now. Her ECG showed atrial rhythm; the PR was shorter than 0.12 s, and the echocardiogram showed LVEDD 43 mm and LVEF 63% (Figure 6). The proband and her sister carried the same three mutations, which were *SCN5A* p.A204E, *TTN* p.I1830T, and *ACTN2* p.M283V, but their clinical phenotypes differed completely.

### Case 5

The proband's niece (III-4) had two mutations, which were *SCN5A* p.A204E and *TTN* p.I18305T. She had syncope and was found to have frequent ventricular premature beats with narrow QRS as a teenager. Her heart was borderline enlarged at that time. She took metoprolol for a few years and then stopped. She suffered syncope again when she was 20. The ECG showed a threshold heart rate and polymorphic premature ventricular contractions. Her current heart size is still in the normal range (LVEDD, 50 mm; LVEF, 55%). She is being followed and has good medical compliance. The niece's daughter (IV-1) carries the same gene mutation as her mother. She is still a toddler and has no obvious symptoms.

## Discussion and Conclusions

Some studies have examined the association between genetic variants and DCM phenotypes (5) which may affect patient clinical prognosis differently. The genetic background of patients with DCM is complex: 7% have a single heterozygous mutation, more than 38% have a compound heterozygous or combined mutation, and 12.8% have three or more mutations (6, 7).

The proband and her sister carried the same three mutations, but her sister did not have heart disease. Various tests showed that her sister's heart function and rhythm were normal. The proband's children developed DCM in childhood. They had identical genetic variants, carrying the same four mutations, which were *SCN5A* p.A204E, *TTN* p.I1830T, *ACTN2* p.M283V, and *PRKAG2* p.D372N. The mutation *PRKAG2* p.D372N was from their father, and the others were from their mother. At the beginning, we thought the father had no family history of related diseases and considered that the variant had little to do with DCM. However, their clinical phenotype was more severe than their mother's, and they both carried *PRKAG2* p.D372N from their father, which might be a modified gene. The *PRKAG2* gene encodes the AMPK γ2 subunit in humans. The mutations of *PRKAG2* can cause *PRKAG2* syndrome. *PRKAG2* syndrome is a rare autosomal dominant disorder characterized by myocardial glycogen storage, myocardial hypertrophy, and ventricular preexcitation. Patients have a high risk of arrhythmias and sudden cardiac death (8). We reviewed the cardiac slides of Case 3 and did PAS staining. Cardiomyocyte hypertrophy and cytoplasmic vacuolization can be seen under the microscope. The vast cytoplasmic glycogen accumulation was confirmed by a positive PAS stain. Although the ACMG guidelines considered the pathogenicity of the *PRKAG2* variant to be “uncertain significance,” these findings further confirmed the *PRKAG2* syndrome. The early onset and severe disease of children might be due to a combination effect of the *SCN5A* p.A204E mutation from their mother and the *PRKAG2* p.D372N mutation from their father.

In the third case, the boy had a heart transplant at the age of 10 years and died suddenly at school 2.5 years postoperatively. No cause of the sudden death was confirmed. Although he had undergone a heart transplant, his paracardial microenvironment might have also been affected. Because the patient did not undergo an endomyocardial biopsy, it was impossible to determine whether he had a chronic rejection reaction. An ECG within 2 years after transplantation showed dynamic changes in T-wave inversion, suggesting ischemia. The underlying molecular genetic mechanisms of the child and environmental factors combined to cause the sudden death. Clinical studies indicate that sudden cardiac death (SCD) is common after heart transplantation. There is no anatomic cause of death in some patients. These deaths are assumed to be primary arrhythmic deaths (9).

It was previously thought that abnormalities in the *SCN5A* gene, which encodes the α subunit of the cardiomyocyte sodium channel, affect the release and utilization of calcium ions in cardiomyocytes *via* the sodium-calcium exchanger, leading to an increased risk for arrhythmias, but with no apparent effect on cardiac anatomy (10). Olson et al. (11) summarized a series of cases with enlarged hearts, pump failure, and a combination of arrhythmias as the main manifestations, and found that *SCN5A* not only caused abnormalities in the electrical activity of the heart but also caused heart enlargement and mechanical abnormalities, which lead to heart failure. The literature suggests that dimers are formed by interactions between the sodium channel α subunits.

Patients with DCM combined with *SCN5A* mutations are prone to ventricular tachycardia, atrioventricular block, and supraventricular tachycardia, and have a higher mortality rate, with life-threatening ventricular arrhythmias occurring at LVEF <35%, also known as arrhythmogenic DCM (AR-DCM) (12). Patients with AR-DCM should be evaluated carefully and systematically by monitoring for arrhythmia. A family history of ventricular arrhythmias often indicates a poor prognosis and an increased risk for SCD (13). The patients in this family had a variety of clinical presentations, but the ECG waveforms of their premature ventricular beats were very similar. The ECG showed bidirectional ventricular tachycardia, multiple sources of premature ventricular beats, and alternating right and left bundle branch blocks, suggesting that the location of the lesion originated from multiple sites in the Hippo lineage. The main manifestations in patients with the same *SCN5A* c.611C>A mutation (2) are multifocal ectopic Purkinje-related premature contractions (MEPPC) and non-sustained ventricular tachycardia. MEPPC syndrome is caused by mutations in the *SCN5A* gene, and patients with or without DCM are at significantly higher risk for SCD. When we found the *SCN5A* mutation in many family members, we performed analyses with the bio-information software SIFT (14), PolyPhen2 (15), and MutationTaster (16). All predicted that the mutation might be harmful, and the ClinVar database included the mutation as “likely pathogenic.” Other mutations at the same amino acid (c.611C>T) are pathogenic and associated with Brugada syndrome, as reported in the Human Gene Mutation Database (17). The mutation was not included in The Genome Aggregation Database (gnomAD) and the genotype and disease co-segregation within the family. Electrophysiological studies showed that Nav1.5-A204E mutant channels exhibited a significant leftward shift of 8mV of the activation curve, leading to a larger hyperpolarized window current when compared to wild type. The ACMG guidelines considered its pathogenicity to be “likely pathogenic.” We concluded that this mutation was the genetic basis for the family.

DCM families are characterized by DCM with conduction system abnormalities. The molecular genetic mechanism of DCM is being elucidated with the discovery of more pathogenic genes and their mutated loci. Cardiomyopathy in many families still cannot be explained by the identified pathogenic genes, which implies that there are unknown pathogenic genes in patients with DCM. A monistic etiology still needs to be considered when cardiomyopathy is combined or complicated with arrhythmias. Our current understanding of the genetic factors associated with DCM and their relationship with prognosis remains inadequate, given that genotype–phenotype interactions are the basis for the molecular mechanisms of disease and translational studies. Genetic testing of patients with DCM is important, and guidelines recommend the genetic testing of first-degree relatives of patients with DCM to facilitate earlier identification of potential patients with DCM and early intervention (18). However, available genetic tests for pathogenicity do not yet explain the pathogenesis in all cases, while clinical sequencing currently detects genetic variants in only 40% of patients with DCM (19), and pathogenic mutations that can be confirmed by genotype–phenotype co-segregation currently account for only about 20% of positive genetic results (20).

In conclusion, the *SCN5A* mutation with multiple DCM-related gene mutations caused a variety of phenotypes, including DCM, arrhythmia, and sudden death in this family. The clinical manifestations of the family with the same mutation are diverse. The early and severe onset in children may be the result of a combination of *SCN5A* and *PRKAG2* mutations. This report shows that a similar phenotype caused by the same variant in a different ethnic background is strong evidence of the variant's DCM and arrhythmic causality. In addition, it broadens the spectrum of mutations in the *SCN5A* and *PRKAG2* genes for possible combined pathogenesis. The dosage effect of the two mutations between the genotype and the phenotype needs further research. Gene diagnosis allows the early detection of patients with subclinical DCM and facilitates early interventions to improve a prognosis, which can be more helpful in the diagnosis and research of DCM.

## Data Availability Statement

The original contributions presented in the study are included in the article/supplementary material, further inquiries can be directed to the corresponding author.

## Ethics Statement

Written informed consent was obtained from the individual(s), and minor(s)' legal guardian/next of kin, for the publication of any potentially identifiable images or data included in this article.

## Author Contributions

All authors listed have made a substantial, direct, and intellectual contribution to the work and approved it for publication.

## Conflict of Interest

The authors declare that the research was conducted in the absence of any commercial or financial relationships that could be construed as a potential conflict of interest.

## Publisher's Note

All claims expressed in this article are solely those of the authors and do not necessarily represent those of their affiliated organizations, or those of the publisher, the editors and the reviewers. Any product that may be evaluated in this article, or claim that may be made by its manufacturer, is not guaranteed or endorsed by the publisher.
