# Corrigendum: Case Report: Family Curse: An *SCN5A* Mutation, c.611C>A, p.A204E Associated With a Family History of Dilated Cardiomyopathy and Arrhythmia

**DOI:** 10.3389/fcvm.2022.944834

**Published:** 2022-06-15

**Authors:** Wen Huang, Rui Xu, Ning Gao, Xia Wu, Cong Wen

**Affiliations:** ^1^Department of Medical Ultrasound, Shandong Provincial Qianfoshan Hospital, Shandong University, Jinan, China; ^2^Department of Cardiology, The First Affiliated Hospital of Shandong First Medical University & Shandong Provincial Qianfoshan Hospital, Shandong Medicine and Health Key Laboratory of Cardiac Electrophysiology and Arrhythmia, Shandong University, Jinan, China; ^3^Department of Cardiology, Shandong Provincial Qianfoshan Hospital, Weifang Medical University, Jinan, China

**Keywords:** *SCN5A*, *PRKAG2*, dilated cardiomyopathy, sudden cardiac death, familial case report

In the published article, there was an error regarding the affiliation for Corresponding Author Rui Xu. Instead of “Shandong Medicine and Health Key Laboratory of Cardiac Electrophysiology and Arrhythmia, Department of Cardiology, The First Affiliated Hospital of Shandong First Medical University and Shandong Provincial Qianfoshan Hospital, Jinan, China,” the affiliation should read “Department of Cardiology, the First Affiliated Hospital of Shandong First Medical University & Shandong Provincial Qianfoshan Hospital, Shandong Medicine and Health Key Laboratory of Cardiac Electrophysiology and Arrhythmia, Shandong University, Jinan, China.”

The authors apologize for this error and state that this does not change the scientific conclusions of the article in any way. The original article has been updated.

## Publisher's Note

All claims expressed in this article are solely those of the authors and do not necessarily represent those of their affiliated organizations, or those of the publisher, the editors and the reviewers. Any product that may be evaluated in this article, or claim that may be made by its manufacturer, is not guaranteed or endorsed by the publisher.

